# Incidence of clozapine-induced hematological side effects in a Tunisian population

**DOI:** 10.1192/j.eurpsy.2022.1842

**Published:** 2022-09-01

**Authors:** M. Shiri, A. Ouertani, S. Madouri, U. Ouali

**Affiliations:** 1 Razi hospital, Psychiatry A, Manouba, Tunisia; 2 Razi Hospital, Psychiatry A, manouba, Tunisia

**Keywords:** blood dyscrasias, incidence, clozapine

## Abstract

**Introduction:**

Clozapine is commonly associated with adverse hematological outcomes. However, incidence of blood dyscrasias in the north African population is scarce.

**Objectives:**

The aim of this study was to assess the incidence of hematological side effects in a Tunisian sample of clozapine treated patients.

**Methods:**

We conducted a retrospective longitudinal chart review of 64 patients on clozapine enrolled in our clozapine consultation between January 1, 2000 and September 2020.

**Results:**

Our sample consisted of 15 women (23.5%) and 49 men (76.5%), mean age was 41.34 ±9.32 years. Patients were diagnosed with schizophrenia in 70.3% of the cases, 7 (10.9%) had a bipolar disorder and 12 (18.8%) had a schizoaffective disorder.We found blood dyscrasias in 21 patients (32.8%). Hematological abnormalities were as follow: 2 cases of agranulocytosis, 8 cases of neutropenia, 13 cases of thrombocytopenia, 5 cases of leukocytosis, 5 cases of eosinophilia and 3 cases of anemia. The incidence rate of hematological side effects was 0.1 case/year- person. The mean clozapine dose at the time of onset of the hematological side effect was 309.52 mg/day(range 25-600 mg/day).The median duration of clozapine treatment prior to developing hematological side effects was 119.71±126.52 days.Clozapine discontinuation was decided in 11 cases due to hematological side effects and reintroduced in 9 cases after normalization of blood count.

**Conclusions:**

This study emphasis on the importance of a regular long-term monitoring of blood count to ensure early detection of hematological side effects.

**Disclosure:**

No significant relationships.

